# Early growth response gene mediates in VEGF and FGF signaling as dissected by CRISPR in corpus luteum of water buffalo

**DOI:** 10.1038/s41598-020-63804-z

**Published:** 2020-04-22

**Authors:** Meeti Punetha, Vikrant S. Chouhan, Arvind Sonwane, Gyanendra Singh, Sadhan Bag, Jonathan A. Green, Kristin Whitworth, Mihir Sarkar

**Affiliations:** 1 ICAR-Indian Veterinary Research Institute, Physiology & Climatology Division, Izatnagar, Bareilly, Uttar Pradesh 243122 India; 2 ICAR-Indian Veterinary Research Institute, Division of Animal Genetics, Izatnagar, Bareilly, Uttar Pradesh 243122 India; 3 University of Missouri-Columbia, Division of Animal Science, Columbia, Missouri USA

**Keywords:** Growth factor signalling, Molecular biology

## Abstract

The EGR family comprises of EGR 1, EGR 2, EGR 3 and EGR 4 which are involved in the transactivation of several genes. A broad range of extracellular stimuli by growth factors is capable of activating EGR mediated transactivation of genes involved in angiogenesis and cell proliferation. However, their role in controlling VEGF A and FGF 2 signaling in the CL of water buffalo is not known. The present study was conducted to understand the role of EGR mediated regulation of VEGF A and FGF 2 signaling in buffalo luteal cells. Towards this goal, luteal cells were cultured and treated with VEGF A and FGF 2 and the mRNA expression pattern of EGR family members were documented. The EGR 1 message was found to be up-regulated in luteal cells of buffalo at 72 hours of culture. The functional validation of EGR 1 gene was accomplished by knocking out (KO) of EGR 1 in cultured luteal cells by CRISPR/Cas9 mediated gene editing technology. The EGR 1 KO cells were then cultured and stimulated with VEGF A and FGF 2. It was observed that VEGF A and FGF 2 induced angiogenesis, cell proliferation and steroidogenesis in wild type luteal cells, whereas the response of the growth factors was attenuated in the EGR 1 KO cells. Taken together our study provides evidence convincingly that both VEGF and FGF mediate their biological action through a common intermediate, EGR 1, to regulate corpus luteum function of buffalo.

## Introduction

The corpus luteum (CL) is essential for the pregnancy establishment and its maintenance as it primarily secretes progesterone^1^. Luteal dysfunction in domestic animals results in inadequate progesterone production, poor embryonic development, infertility and increased pregnancy failure in buffalo cow^2^. Although, LH and PGF2α plays important role in development and regression of corpus luteum, but there are several other ovarian peptides which are critical for development, function and regression of CL. These include fibroblast growth factors (FGF)^3^, the vascular endothelial growth factor (VEGF)^4^, thrombospondins (TSPs)^5^, IGF^6^, Leptin^7^, Angiopoietin^8^ and Ghrelin^9^. Among these VEGF helps in the regulation of angiogenesis, vasculogenesis, steroidogenesis of the CL in several species such as cattle^10^, pigs^11^, and buffaloes^12^. The transition of follicular cell to luteal cell, angiogenesis in CL and its function is also controlled by FGF 2 in cattle^13^ and buffalo^3^. Growth factors such as VEGF and FGF family mediate their physiological role by binding with their transmembrane tyrosine kinase receptor. The complex dimerizes and stimulates the receptor’s intrinsic tyrosine kinase activity. Ligand activation of Receptor tyrosine kinase (RTK) activates downstream pathways such as mitogen-activated protein kinase (MAPK), protein kinase B (PKB)/ phosphatidylinositol-3-kinase (PI3K) and protein kinase C (PKC) in a ligand and cell-context manner^14^. The downstream signaling further phosphorylates the dual acceptor motif ERK1/2. Activated ERK translocates into the nucleus and promotes binding SRF/Elk-1 which subsequently upregulates EGR 1^15–17^.

FGF signaling showed the transcriptional abundance of NR4A1 and EGR 1 in granulosa cells ^18,19^. FGF 1, FGF 2 and VEGF are potent EGR genes activators ^15,20–22^. Early growth response genes are key transcriptional regulators which are highly associated with cell growth, survival ^23,24^ and apoptosis^25^. The EGR family mainly comprises of four transcriptional factors which includes EGR 1, EGR 2, EGR 3 and EGR 4. Among these transcriptional factors EGR 1, 2 and 3 are activators, whereas EGR 4 acts as a repressor^26^. EGR proteins bind to GC-rich DNA recognition sites and turn on the expression of different genes required for differentiation and mitogenesis in different tissues^27^. EGR 1 is also recognized as NGFI-A, KROX24, ZIF268, and TIS8 which is an inducible transcription factor that belongs to a family of immediate early response genes ^28,29^. EGR 1 is inducibly expressed in many different cell types including endothelial cells in mice^30^; granulosa cell in bovines^31^ and luteal cells in bovines^32^. However, depending on cell type, EGR 1 acts as both a transcriptional activator and repressor^33^. EGR 1 when binds to DNA it modify gene transcription via its both coactivators and corepressors dependent mechanisms. Since, EGR 1 controls the expression of a wide variety of genes it act as a master regulator^23^. FGF 1, FGF 2 and VEGF are potent EGR genes activators ^15,20–22,34^. The up regulation of EGR 1 by FGF 2 in endothelial cells suggests its role in inducing proangiogenic factors^35^. Compelling evidences are provided by Santigo^36^ which suggests that fibroblast growth factor (FGF 2) should be embodied in the list of critical mediators regulating EGR 1 expression. EGR 1 null mice exhibit impaired reproductive function which shows that EGR 1 is important for the normal functioning of ovaries^37^. EGR 1 plays a central role in regulation of rapid transition of follicular cells to luteal cells in rats^38^. According to Liu *et al*.^39^, EGR 3 is strongly up regulated by VEGF in human endothelial cells. Various stimuli such as hormones and growth factors also up regulates EGR 3 like EGR 1^40–42^.

Although extensive efforts have been made to delineate the mechanisms by which VEGF A and FGF 2 induces angiogenesis, steroidogenesis and cell survivability in murine and human endothelial cells, but no information could be traced to date to understand the insight of EGR mediated regulation of VEGF and FGFs signaling in buffalo luteal cells. Hence, we hypothesized that EGR protein(s) could act as mediators of VEGF and FGFs signaling in bubaline luteal cells. Thus, the present study was aimed to explore the functional role of EGR family members on VEGF and FGF signaling in the luteal cells of water buffalo.

## Methods

All methods and experimental protocols were carried out in accordance with relevant safety guidelines and regulations.

### Experiment 1

To test whether there is any pairing of expression of EGR genes with VEGF and FGF signaling in bubaline luteal cells.

### Collection of CL

Ovaries from healthy buffalo cows with normal reproductive tracts were collected in 1X phosphate-buffered saline (PBS) at 37 °C in a vacuum flask from a local abattoir. In the present experiment, only mid luteal stage corpus luteum was used. The selection of mid stage CL was done according to the pre-estabished protocol^43^.

### Tissue collection, luteal cell culture and treatment with VEGFA and FGF2

**Figure 1:**
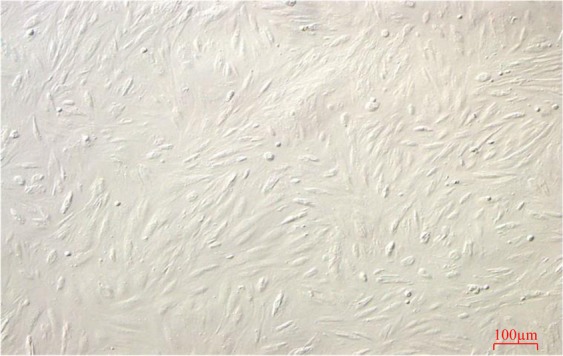
Culture of attached luteal cells (10X magnification).

The luteal cells were cultured using the pre-established protocol^44^. In brief, mid stage CLs were removed from the ovary with all connective tissue; and CLs were then sliced up using BP blades (Bard-Parker Surgical Blade). The minced luteal tissue was washed three times at 150 *g* for 5 min at room temperature with the washing medium containing Dulbecco’s modified Eagle’s medium (DMEM)/F12 medium (Hyclone) and antibiotic–antimycotic solution. The minced cells including luteal, endothelial, pericytes and fibroblasts were then digested by incubating the luteal tissue in (DMEM)/F12 medium containing collagenase, DNase I and bovine serum albumin (BSA) for 2 ×45 min in an orbital shaker incubator at 37 °C. The cells were then filtered through a 70 µm filter and after washing were resuspended in culture medium containing DMEM/F12 medium, 10% fetal bovine serum (FBS; Sigma-Aldrich) and 1% antibiotic–antimycotic solution. The cell viability was determined by Trypan blue vital stain. The cells were then plated out at 1.5 ×10^5^ viable cells per well in a 24-well plate with 1 mL culture medium in a humidified CO2 (5%) incubator at 37 °C. The cells were allowed to attach and grow (75–80% confluent) for 72 hrs (Fig. 1). There were four experimental conditions: (i) Wild type luteal cells; ii) Luteal cell treated with VEGF A @ 100 ng/ml^3^; (iii) Luteal cell treated with FGF 2 @50 ng/ml^3^. Cells were cultured for 72 hrs in replicates of six for each group. Spent media was stored at −20 °C until required for P_4_ RIA and cells were harvested for the total RNA isolation and qPCR analysis to determine the expression pattern of EGR 1, EGR 2, EGR 3, EGR 4, 3β HSD, STAR, CYP11A1, vWF, AKT and BAX genes (Table 1). To assess the cell proliferation and apoptosis MTT and Annexin V apopotosis assay was performed.

**Table 1:** Target gene, primer sequences and amplicon length used in the qPCR and Knockout study.

Gene	Sequences of nucleotide (5′−3′)	Amplicon length (bp)	EMBL accession no.or reference
EGR 1	Forward: AGCTGTGCAGTGCAGTCCAACGAC Reverse: TAGTCGGGGATCATGGGAACCTG	194	XM_006070841.2
EGR 2	Forward: TCCCCAATGCCGAACTGGGA Reverse: GTCAATGGAGAACTTGCCCATGTA	182	>XM_025284605.1
EGR 3	Forward: TCGGTCTGACCAACGAGAAGCCCA Reverse: GATGTTGTCCTGGCACCAGTTGGA	137	>XM_006040943.2
EGR 4	Forward: GACGAGGAACTGCCACCTCC Reverse: CTCACAGACCTAGATGCTCGGA	103	>XM_006045927.2
vWF	Forward: ATCGTAGGGGACTTCCAAGGTGG Reverse: CGGTCTCCAGGTATAGCCCTCTGG	154	Mishra *et al*., 2016
AKT	Forward: AAACCGTTACCTTGCTATG Reverse: TGCCCAGTTCGTTTCAGT	159	NM_174056. 3
BAX	Forward:AACATGGAGCTGCAGAGGAT Reverse: CAGTTGAAGTTGCCGTCAGA	104	>XM_025269476.1
RPS15A	Forward:AGGGCTGGGAAAATTGTTGTGAA Reverse: TGAGGGGATGGGAGCAGGTTAT	104	Paul *et al*., 2018
CYP11A1	Forward: AGACTTGGAGGGACCATGTAGC Reverse: TGCCTGGGTAATTCCTAAATTC	117	>XM_025271874.1
3βHSD	Forward: AATCCGGGTGCTAGACAAAGT Reverse: CACTGCTCATCCAGAATGTCTC	111	>XM_006049357.2
StAR	Forward: CTGCGTGGATTAACCAGGTTCG Reverse: CCAGCTCTTGGTCGCTGTAGAG	84	XM_006054485.2
EGR1 SgRNA	Forward:TAATACGACTCACTATAGGTCCATGGTGGGCGAATG Reverse: TTCTAGCTCTAAAACCATTCGCCCACCATGGAC	—	—
EGR1 Genomic Detection	EGR1 For1: CTACCCCAGCCTCGGTAGCA EGR1 Rev1: TCAGGTGCTCGTAGGGCTGC		

### Experiment 2

To determine the effect of modulating EGR expression on VEGF and FGF target response in bubaline luteal cells

### Production of EGR 1 knock out (KO) luteal cells

**Figure 2:**
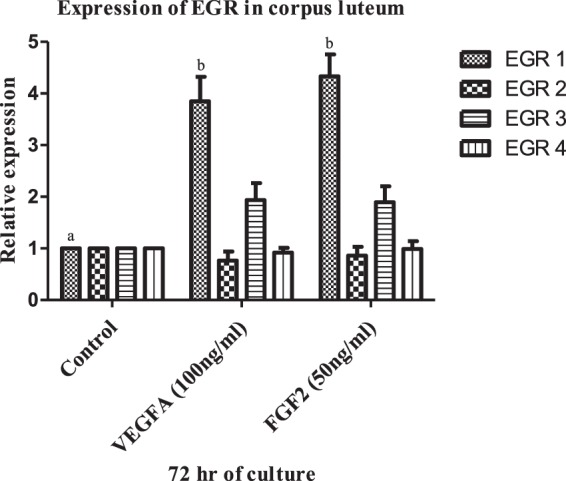
mRNA expression of EGR in MLC treated with VEGF A 100 ng/ml and FGF 2 50 ng/ml at 72 hrs. All values are shown as mean ± SEM. Different superscripts denote statistically different values (P < 0.05).

Based on the results of Experiment no.1, expression of EGR 1 was found to be up regulated by VEGF A and FGF 2 (Fig. 2) and hence it was chosen to knock out by CRISPR/Cas9 gene editing tool. In this approach, components of CRISPR/Cas9 system (single guide RNA and Cas9) were delivered into the bubaline luteal cells via lipofection and the details of the methodology used are as follows.

### Preparation of synthetic single guide RNA (SgRNA)

EGR 1 gene specific guide (20 nt) was designed in silico by using available software like CRISPOR (Table 1). For SgRNA synthesis, the T7 promoter sequence was added to SgRNA template and the *in vitro* transcription (IVT) template was then generated by polymerase chain reaction (PCR) amplification using designed primers. Briefly, SgRNA DNA template was assembled by PCR using the Phusion High-Fidelity PCR Master Mix (Invitrogen, Cat. No. A29377) in a thermocycler followed by generation of SgRNA by *in vitro* transcription using the Transcript Aid Enzyme Mix. Later the *in vitro*-transcribed SgRNA was purified using purification columns kit according to the manufacturer’s instruction. Aliquots from an IVT reaction were separated on an agarose gel to assess the quality of the reaction and the concentration of purified SgRNA was measured by Nanodrop spectrophotometer.

### Transfection and genomic cleavage detection

Cultured luteal cells with 30–70% confluency were transfected with Cas9-gRNA RNP by using the Lipofectamine CRISPRMAX Transfection Kit (Invitrogen; Cat. No. CMAX00015) as per the manufacturer’s protocol. Ready-to-transfect wild-type Cas9 protein (GeneArt Platinum Cas9 Nuclease; Cat. No. B25640) was procured from Invitrogen. Briefly, Cas9-gRNA RNP was prepared in one tube and CRISPRMAX reagent diluted in Opti-MEM medium (Cat. No. 11058‐021) in another tube was incubated for 5 min at room temperature. The solution from the tubes was intermixed and incubated further for 10 min and was transfected to luteal cells. Validation of EGR 1 KO was performed using a GeneArt Genomic Cleavage Detection Kit (Invitrogen; Cat No. A24372) as per the manufacturer’s instructions. The cleavage efficiency of the EGR 1 knock out was calculated by the following formulae^45^.

$${\rm{Cleavage\; efficiency}}=[({\rm{sum\; of\; cleaved\; band\; intensities}})/({\rm{sum\; of\; cleaved\; and\; parental\; band\; intensities}})]\times 100 \% $$

### Treatment of EGR 1 KO luteal cells with VEGFA and FGF2

EGR 1 KO luteal cells were then treated with similar dose of VEGFA and FGF2 as in Experiment no.1 and cultured for 72 hrs in replicates of six in DMEM/F12 for each treatment group. There were five experimental conditions: i) EGR 1 KO luteal cells without treatment; ii) Wild type cells without treatment; iii) EGR 1 KO luteal cells with treatments of VEGFA @ 100 ng/ml; iv) EGR 1 KO luteal cells with treatments of FGF2 @ 50 ng/ml. After 72 hrs of culture, cells were collected from culture plates. The spent media was stored at −20 °C until required for P4 immunoassay and cells were harvested for total RNA isolation and qPCR analysis was performed to determine the expression pattern of EGR 1, 3βHSD, STAR, CYP11A1, vWF, AKT and BAX gene (Table 1).

### Cell viability assays

The wild type luteal cells and EGR 1 KO luteal cell were seeded on 96-well plates and incubated at 37 °C for 72 hr with media without treatment and with media containing VEGF A and FGF 2 (100, 50 ng mL^−1^). Subsequently 20 µl of MTT solution (5 mg/ml in PBS) 3-(4,5-dimethylthiazole-2-yl)-2,5-diphenyltertrazolium bromide (MTT; MP Biomedical) was added to each well and was incubated for 4 hr at 37 °C. Supernatant was then removed and 100 mL dimethyl sulphoxide (DMSO; MP Biomedical) was added and absorbance at 450 nm was detected with a microplate reader (Biorad).

### Apoptosis assay

The differentiation between the apoptotic and healthy cells was performed by Annexin V apoptosis detection kit (BD bioscience). Both wild type and EGR 1 knock out luteal cells were cultured at 37 °C for 72 hrs with media without treatment and with media containing VEGF A and FGF 2 (100, 50 ng mL^−1^). The cells were trypsinized using 0.05% Trypsin/EDTA solution and were harvested and treated with 5 μl of both annexin V and PI according to manufacturer’s instruction. The apoptotic signal was detected by Axio Observer.Z1 (Carl Zeiss Micro Imaging GmbH, Germany) microscope.

### Primers

The primers *EGR 1*, *EGR 2*, *EGR 3*, *EGR 4*, *AKT*, *BAX*, *StAR*, *3βHSD* and *CYP11A1* were designed using DNAStar (online trial version, DNASTAR Lasergene 6, 2004), Gene Tool (online trial version, 2004) and Oligo Analyser 3.1 (open access tool, 2017) software. Published primers were used for 40 S ribosomal protein S15 (*RPS15A*)^44^ and *vWF*^46^. Details of the primers used are presented in Table 1.

### Quantitative qPCR analysis

Isolation of total RNA from cultured luteal cells was done by QIAzol reagent (QIAGEN) according to the manufacturer’s instructions. The quality of RNA was determined via A260/A280 in Nanodrop spectrophotometer. Integrity of the total RNA was verified by agarose gel electrophoresis. Total RNA (1 µg) from different samples were reverse transcribed to cDNA using a RevertAid First cDNA synthesis kit (ThermoFisher Scientific) according to the manufacturer’s instructions by using oligo dT primers at 42 °C for 60 min. Quantitative real-time PCR was performed using the Maxima SYBR Green qPCR kit (Thermo Scientific). Each sample was run in triplicate in 25 µL reaction mixture, which consisted of 12.5 µL SYBR green mix, 0.5 µL each of 0.3 µM forward and 0.3 µM reverse primers, 1 µL cDNA and 10.5 µL nuclease-free water. The following general qPCR protocol was followed: initial denaturation at 95 °C for 10 min followed by 40 cycles of denaturation at 95 °C for 15 s, annealing and extension at 60 °C for 60 s. The efficiency of Real-time PCR was determined by amplification of a standardized dilution series and slopes.

### Hormone determination

Progesterone (P4) concentration in the spent media of cultured luteal cell was estimated by P4^125^I RIA kit (Immunotech) as per the manufacturer’s instructions. The intra and inter-assay coefficients of variation were 6.5% and 7.2% respectively.

### Statistical analyses

All experimental data are shown as mean ± SEM. The statistical significance of differences in mRNA expressions of all genes, P4 concentrations in spent culture media and % cell viability was assessed using the software SPSS.22 (online trial version) by one-way analysis of variance followed by Tukey’s honestly significant difference (HSD) test as a multiple comparison test. Differences were considered to be significant at *p* < 0.05.

## Results

### Effect of VEGF A and FGF 2 on expression of Early Growth Response gene in CL

It was observed from the analysis that VEGF A (100 ng/ml) and FGF 2 (50 ng/ml) treatment significantly (*p* < 0.05; Fig. 2) up regulated EGR 1 gene expression in luteal cells of buffalo at 72 hr. However, the relative expression of other members of EGR family viz. EGR 2, EGR 3 and EGR 4 remained unaffected by the VEGF A and FGF 2 treatment.

### Effect of EGR 1 on luteal cell angiogenesis in buffalo

**Figure 3:**
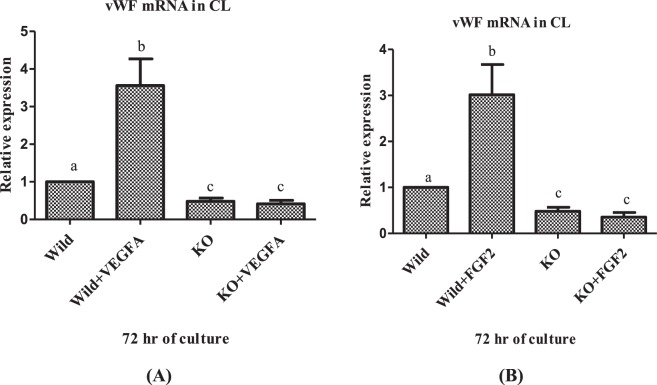
mRNA expression of vWF in MLC and EGR 1 KO MLC treated with VEGF A 100 ng/ml and FGF 2 50 ng/ml at 72 hrs. All values are shown as mean ± SEM. Different superscripts denote statistically different values (P < 0.05). EGR 1 KO, Early Growth Response factor 1 Knock out; MLC, Mid stage luteal cells.

**Figure 4:**
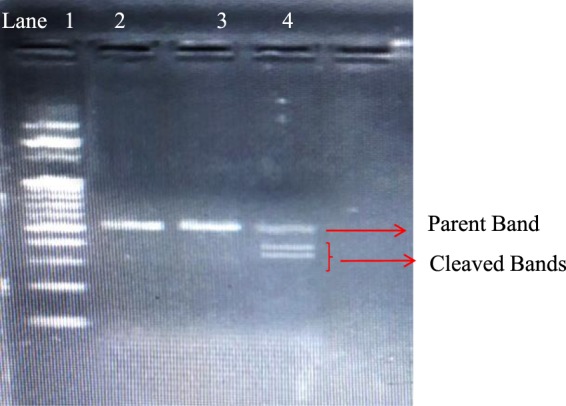
Gel image of genomic cleavage detection assay of luteal cells transfected with Cas9 and EGR 1 SgRNA using Lipofectamine 2000. Lane 1, 100 bp DNA ladder; Lane 2, negative control sample with intact EGR 1 gene; Lane 3 sample without T7/E1 enzyme digestion, Lane 4: Sample, showing parent and both the cleaved bands after addition of T7/E1 enzyme.

The effect of VEGF A and FGF 2 showed significant up regulation (*p* < 0.05; Fig. 3A,B) on the expression of the von Willebrand factor (vWF) in treated wild type luteal cells when compared with the wild type luteal cells without treatment. In order to examine the functional role of EGR 1 on luteal cell angiogenesis in water buffalo, EGR 1 was knocked out via CRISPR/Cas 9 genome editing technology (Fig. 4). The present study revealed a significant down regulation in the expression of endothelial cell marker, vWF(*p* < 0.05; Fig. 3A,B) in EGR 1 KO luteal cells when compared with the wild type luteal cells. Further, to determine the functional role of EGR 1 on VEGF A and FGF 2 induced angiogenesis, the EGR 1 KO luteal cell were treated with VEGF A (100 ng/ml) and FGF 2 (50 ng/ml) over a period of 72 hour. There was a significant (*p* < 0.05; Fig. 3A,B) difference in the expression of vWF in EGR 1 KO luteal cell with VEGF A and FGF 2 treatment when compared with the wild type luteal cells with treatment. Thus, the present finding confers the angiogenic role of EGR 1 in luteal cells of buffalo.

### Effect of EGR 1 on luteal cell viability and function in buffalo

**Figure 5:**
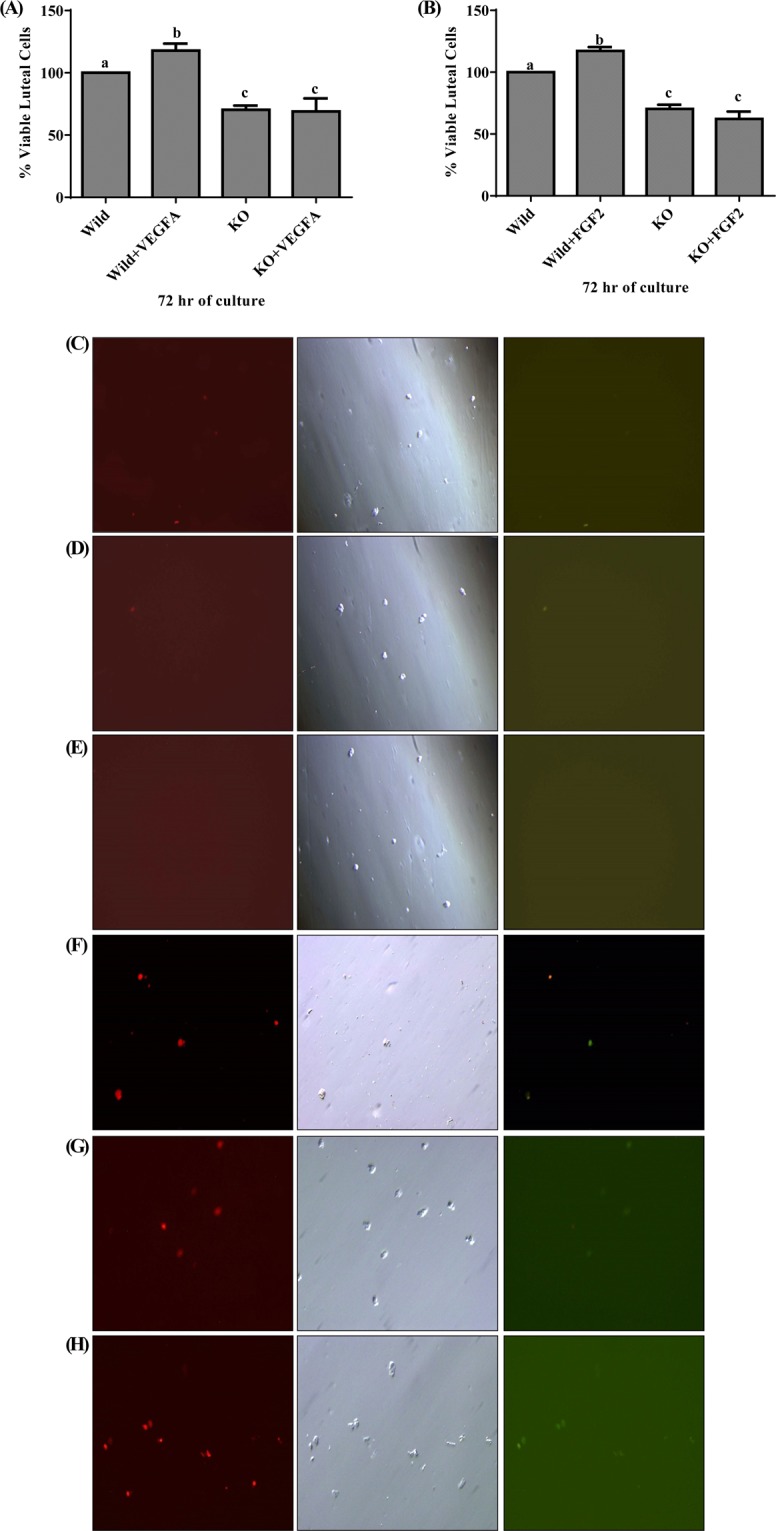
Demonstration of percentage viable n MLC and EGR1 KO MLC treated with VEGF A 100 ng/ml and FGF 2 50 ng/ml at 72 hrs (**A**,**B**). Images of wild type luteal cells (**C**) Wild type luteal cells with VEGF A (**D**); Wild type luteal cells with FGF 2 (**E**); EGR 1 KO luteal cells (**F**); EGR 1 KO luteal cells with VEGF A (**G**); Wild type luteal cells with FGF 2 (**H**) showing cells under bright field; Green fluorescence, signal for FITC Annexin; Red fluorescence, signals for Propidium iodide. All the groups of cells were cultured for 72 hour followed by trypsinization and treatment with Annexin V apoptosis detection and Propidium iodide dye.

**Figure 6:**
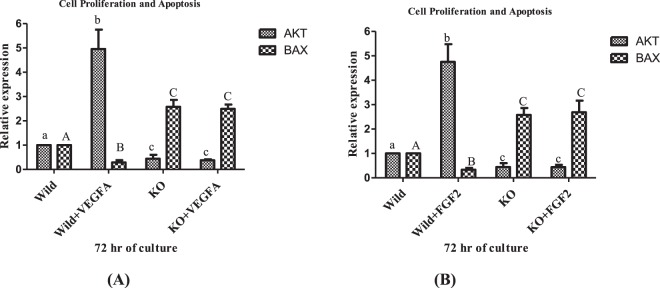
mRNA expression of AKT and BAX in MLC and EGR1 KO MLC treated with VEGF A 100 ng/ml and FGF 2 50 ng/ml at 72 hrs. All values are shown as mean ± SEM. Different superscripts denote statistically different values (P < 0.05). EGR 1 KO, Early Growth Response factor 1 Knock out; MLC, Mid stage luteal cells.

To understand the effect of VEGF A and FGF 2 on luteal cell viability, MTT assay was performed. The MTT study revealed significant difference in viability between wild type luteal cells and wild type luteal cell treated with VEGF and FGF 2 (*p* < 0.05; Fig. 5A,B). The effect of VEGF A and FGF 2 on viability of luteal cells was supported by Annexin V-FITC/PI analysis. After 72 hr incubation, very less green and red fluorescent signals for FITC Annexin and Propidium iodide were detected in wild type luteal cells with treatment compared to that of wild type cells without treatment which shows lesser number of apoptotic and dead cell (Fig. 5C–E) under Axio Observer.Z1 (Carl Zeiss Micro Imaging GmbH, Germany) microscope. Again mRNA expression study of AKT and BAX was done to confirm the effect of VEGF A and FGF 2 in cell proliferation and apoptosis. The study revealed significant up regulation in AKT expression and significant down regulation in BAX (*p* < 0.05; Fig. 6A,B) expression in wild type treated luteal cells when compared with wild type luteal cells without treatment.

For understanding the effect of EGR 1 on luteal cell viability and the effect of EGR 1 on VEGF A and FGF 2 induced cell proliferation, MTT assay was performed on EGR 1 KO luteal cells and EGR 1 KO luteal cells with VEGF A and FGF 2 treatment. The study showed significant difference in the viability of EGR 1 KO luteal cells and EGR 1 KO luteal cell with VEGF A and FGF 2 treatment in comparison to the wild type luteal cells with and without treatment. There were almost 70% viable EGR 1 KO luteal cells, 68% viable EGR 1 KO luteal cells with VEGF A treatment and 62% viable EGR 1 KO luteal cell with FGF 2 treatment (*p* < 0.05; Fig. 5A,B). The effect of EGR 1 on viability was also checked by Annexin V-FITC/PI analysis. After 72 hr incubation, fluorescent signals detected in EGR 1 KO luteal cells and EGR 1 KO luteal cells with VEGF A and FGF 2 treatment were more than wild type luteal cells without treatment and wild type luteal cells with treatment which shows higher number of dead and apoptotic EGR 1 KO cells (Fig. 5F–H). The mRNA expression study of AKT and BAX in EGR 1 KO luteal cells and EGR 1 KO luteal cells with VEGF A and FGF 2 treatment showed significant difference (*p* < 0.05; Fig. 6A,B) with wild type luteal cells and wild type cells with treatment. These findings indicate that EGR 1 contributes to luteal cell survival and has anti-apoptotic role in VEGF A and FGF 2 signaling in corpus luteum of buffalo.

### Effect of EGR 1 on steroidogenesis in luteal cell of buffalo

**Figure 7:**
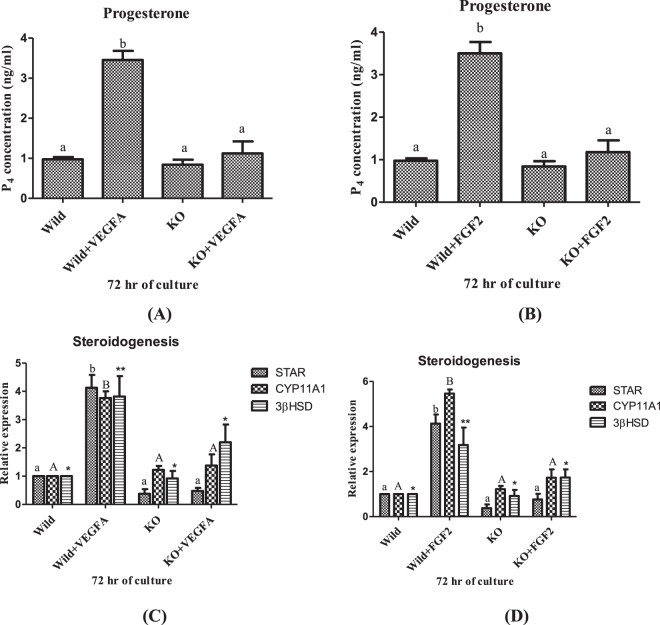
Progesterone concentration in MLC and EGR1 KO MLC (**A**,**B**) and mRNA expression of StAR, CYP11A1 and 3βHSD in MLC and EGR 1 KO MLC treated with VEGF A 100 ng/ml and FGF 2 50 ng/ml at 72 hrs (**C**,**D**). All values are shown as mean ± SEM. Different superscripts denote statistically different values (P < 0.05). EGR1 KO, Early Growth Response factor 1 Knock out; MLC, Mid stage luteal cells; 3βHSD, 3beta hydroxyl steroid dehydrogenase; CYP11A1, cytochrome P450 side chain cleavage subfamily A1; StAR, steroidogenic acute regulatory protein.

To determine the steroidogenic role of VEGF A and FGF 2, the progesterone concentration in the spent media of cultured wild type luteal cells and wild type luteal cells with VEGF A and FGF 2 treatment was measured. The treated wild type luteal cells showed significant up regulation (*p* < 0.05; Fig. 7A,B) in progesterone concentration as compared to the wild type luteal cells without treatment. The relative expression of enzymes controlling the progesterone biosynthetic pathway including StAR, CYP11A1 and 3βHSD was also analysed. The wild type luteal cells with treatment showed significant upregulation of StAR, CYP11A1 and 3βHSD expression in comparison to wild type luteal cells without treatment (Fig. 7C,D). Thus, confirming the steroiodgenic role of VEGF A and FGF 2 in luteal cells of buffalo over a period of 72 hrs.

The effect of EGR 1 on steroidogenesis and its pathway was also studied. The progesterone concentration in the spent media of EGR 1 KO luteal cells did not show any significant difference with the wild type luteal cells (Fig. 7A,B). The expression of steroidogenic enzymes also remains unaffected in the EGR 1 KO luteal cells. However, the EGR 1 KO luteal cells with treatment showed significant difference (*p* < 0.05; Fig. 7C,D) in progesterone concentration, StAR, CYP11A1 and 3βHSD with the wild type luteal cells with treatment. Thus, the present finding is convincing enough to demonstrate the novel role of EGR 1 in the steroidogenic response to VEGF A and FGF 2 signaling.

## Discussion

Early growth response gene stimulates one or more genes which serves as a transcription factor and activates cell-specific response of the genes in order to carry out the ultimate response of the original stimulus. The best known early growth response genes include c-jun, c-fos, and EGR 1. EGR 1 regulates diverse functions including some related to ovarian responses of mitogens, growth factors etc^33^. It plays a key regulatory role via inducing the expression of genes directing the effector stimuli^23^. The role of EGR 1 was elucidated in a variety of pathophysiological responses^47^. In the present study, the relevance of EGR 1 in response to VEGF A and FGF 2 signaling to ovarian physiology has been demonstrated. To the best of our knowledge it is the first study to understand the insight of Early Growth Response genes mediated regulation of FGF 2 and VEGF A signaling in buffalo luteal cells. In the current study, we reported that VEGF A and FGF 2 significantly up regulates EGR 1 mRNA expression (Fig. 2) in wild type luteal cells and causes an EGR 1 dependent transcriptional activation. Previous studies reported the expression of EGR 1 in corpora lutea of cattle ^32,48^. The mRNA expression of EGR 1 can be enhanced by hypoxia, certain hormones and growth factors like bFGF and VEGF in cultured endothelial cells ^49,50^. FGF 1, FGF 2 and VEGF are potent EGR genes activators^34^. EGR 3 is found to have an important downstream role in angiogenesis of VEGF stimulated endothelial cell^26^. Various stimuli such as hormones and growth factors also up regulates EGR 3 like EGR 1^40–42^.

EGR 1 plays a pivotal role in acting as a convergence point for multiple signaling pathways^51^. VEGF A and FGF 2 stimulates EGR 1 gene by the MAPK signal transduction pathway in many cell types^52^. According to Santiago *et al*.^36^. FGF 2 is an important mediator which regulates the expression of EGR 1 following injury in endothelial cells. FGF signaling increased EGR 1 mRNA levels in bovine granulosa cells^18,19^. VEGF induces transcriptional abundance of EGR 1 when treated in human microvascular endothelial cells^53^ and in the uterine stromal cells^54^.

Angiogenesis is a complex multistep system which includes degradation of endothelial basement membrane, cellular proliferation, canalization, branching and maturation of vessels. Angiogenesis in corpus luteum is regulated by a number of growth factors including IGF^6^, FGF 2^3^, and VEGF A^55^. VEGF A and FGF 2 mediate its biological role via binding with trans-membrane tyrosine-kinase receptors, i.e. Flt-1 (VEGFR1) and KDR (VEGFR2) for VEGF ^56,57^ and FGFR1 to FGFR4 for FGF^58^. Ligand activation of Receptor tyrosine kinase (RTK) activates MAPK signaling which then phosphorylates ERK1/2 which translocates into nucleus and promotes binding of SRF/Elk-1, which subsequently up regulates EGR 1^52^. EGR 1 is described as the key mediator involved in a wide range of vascular functions^59^. In order, to validate the transcriptional and functional role of Early Growth Response gene on VEGF and FGF signaling in bubaline luteal cells we have attempted to knockout EGR 1 gene from buffalo luteal cells via a very efficient CRISPR/Cas9 genome editing tool. Previous studies reported that inhibition of EGR 1 inhibits angiogenesis in subcutaneous Matrigel plugs in mice^30^. We demonstrated significant down regulation of vWF in both the EGR 1 KO luteal cells and EGR 1 KO luteal cell treated with FGF 2 and VEGF A when compared with wild type luteal cells with and without treatment. EGR 1 on activation binds with the GC-rich elements of the promoter regions of genes which are involved in angiogenesis^23,30^. VEGF induced EGR 1 mediated gene transcription is an important mechanisms for angiogenesis and vasculogenesis processes^60^. The up regulation of EGR 1 in FGF 2 treated endothelial cells is considered to play an important role in inducing proangiogenic factors^35^. In another study, EGR 1 has been found additionally engaged with angiogenesis in mouse^61^.

VEGF A and FGF 2 both play a pivotal role in acting as a cytoprotective factor and inhibiting apoptosis in order to stimulate luteal cell survival ^3,4^. In the present study, the knocking out of EGR 1 in luteal cells of buffalo showed decrease percentage of cell viability as compared to wild type luteal cells suggesting pro survival role of EGR 1 in luteal cells which can also be confirmed by significant up regulation of BAX and down regulation of AKT in EGR 1 KO cells. When the knock out luteal cell were treated with VEGF A and FGF 2, the percentage of cell viability remained similar to knock out cells without treatment indicating EGR 1 may serve as an important mediator for VEGF A and FGF 2 mediated signaling pathway for cell proliferative responses. Induction of EGR 1 via growth factors in a variety of tissues and cells is immersed in cellular differentiation and proliferation^30^. EGR 1 protein regulates the expression of genes critical to cell proliferation through direct or indirect mechanism^62^. EGR 1 has emerged as an important regulator of cellular physiology due to its ability to modulate the expression of cell cycle regulatory proteins ^63,64^. The embryonic stem cells lacking EGR 1 shows impaired proliferation and differentiation^65^. EGR 1 is highly associated with cell growth, survival^24^ and apoptosis^25^. EGR 1 is a cell proliferation-promoting protein and is also an active part of the apoptotic-signaling cascade, suggesting that it has both pro-survival and pro-apoptotic activities depending on cell context and the primary stimulus^47^.

VEGF A and FGF 2 play an important role in luteal cell steroidogenesis^3,4^ which was concluded by the significant up regulation (*p* < 0.05) of 3βHSD, StAR, and CYP11A1 mRNA expression and P4 estimation in the spent media of wild type luteal cells with VEGF A and FGF 2 treatment. In the present study, our results showed that EGR 1 knock out luteal cells did not show significant difference in the P4 concentration and steroidogenic enzyme expression as compared to the wild type luteal cells. However, the KO luteal cells with treatment showed significant down regulation of expression of key steroidogenic enzyme in comparison to the wild type luteal cells with treatment. In mammals mitogen-activated protein kinase signaling is responsible for the regulation of ovarian steroidogenesis^66^. The endothelial cells when treated with VEGF A leads to significant up regulation of EGR 1 through activation of MEK/ERK module of MAP^67^. VEGF and FGF 2 activates downstream signaling pathway involving the mitogen-activated protein kinase (MAPKs) which subsequently converges into EGR 1 promoter^35^ and the knocking out of EGR 1 led to decreased steroidogenesis. The previous study reported that EGR 1 did not change the progesterone secretion or StAR transcripts expression in bovine granulosa cell^31^. EGR 1 protein mediates development of follicular and luteal cells. EGR 1 is significant for placental angiogenesis^54^, embryo implantation and epithelial-stromal cross-talks in the uterus^68^. In this way, EGR 1 is considered significant for reproductive function and development^69^. The role of EGR 1 in reproduction was accentuated in genetic studies which has shown that EGR 1 ablated mice were found to be infertile^37^. Thus, the present study suggests that VEGF A and FGF 2 induced EGR 1 mediated gene transcription is an important mechanisms for steroidogenesis in luteal cells of buffalo.

The results of the present study provide important insights about EGR 1 gene regulation. Our finding confirms that the transcriptional abundance of EGR 1 is induced by VEGF A and FGF 2 and provides a potential link in VEGF and FGF 2 signaling in the cultured luteal cells of buffalo.

Growth factors are considered an essential protein in cell signaling which binds with specific cell surface receptors which further activates and coordinates complex regulatory network functions including cellular proliferation, growth, maturation and their function. Accordingly, they are recognized as beneficial tools in research and biopharmaceutical development with innumerable potential applications. It has been established by many research investigations that two important growth factors viz. VEGF A and FGF 2 regulate ovarian function to a greater extent alone or in combination in water buffalo. Findings of our study suggest that both the growth factors operate through a common intermediate i.e. EGR1 and this paves the way for potential therapeutic application of this identified intermediate in amelioration of reproductive problems in water buffalo.

## Conclusion

In the present study, we have demonstrated that VEGF A and FGF 2 significantly up regulates EGR 1 in luteal cells of buffalo and initiates EGR 1 dependent transcriptional activation. The knock out of EGR 1 via CRISPR/Cas9 attenuated VEGF A and FGF 2 induced luteal cell angiogenesis, proliferation and steroidogenesis and also increased apoptosis. The present results characterize the functions of EGR 1 in the regulation of luteal cells angiogenesis, cell proliferation and steroidogenesis and as well as provide important insights into the molecular mechanisms that mediate VEGF A and FGF2 signaling. These results suggest that EGR 1 may function as a part of a multifunctional class of master and control gene regulators.
